# *In Vitro* Gene Expression Dissected: Chemostat Surgery for *Mycobacterium Tuberculosis*

**DOI:** 10.1002/cfg.184

**Published:** 2002-08

**Authors:** Brian W. James, Joanna Bacon, Tobias Hampshire, Kim Morley, Philip D. Marsh

**Affiliations:** Centre for Applied Microbiology and Research Salisbury, Wiltshire SP4 0JG UK

## Abstract

A unique approach, combining defined and reproducible *in vitro* models with DNA microarrays, has been developed to study environmental modulation of mycobacterial gene expression. The gene expression profiles of samples of *Mycobacterium tuberculosis*, from independent chemostat cultures grown under defined and reproducible conditions, were found to be highly correlated. This approach is now being used to study the effect of relevant stimuli, such as limited oxygen availability, on mycobacterial gene expression. A modification of the chemostat culture system, enabling largevolume controlled batch culture, has been developed to study starvation survival. Cultures of *M. tuberculosis* have been maintained under nutrient-starved conditions for extended periods, with 10^6^ – 10^7^ bacilli surviving in a culturable state after 100 days. The design of the culture system has made it possible to control the environment and collect multiple time-course samples to study patterns of gene expression. These studies demonstrate that it is possible to perform long-term studies and obtain reproducible expression data using controlled and defined *in vitro* models.


**Comparative and Functional Genomics**
*Comp Funct Genom* 2002; **3**: 345–347.
Published online in Wiley InterScience (www.interscience.wiley.com). **DOI:** 10.1002/cfg.184

**Conference Review**

### *In vitro* gene expression dissected: chemostat surgery for *Mycobacterium tuberculosis*

Brian W. James, Joanna Bacon,* Tobias Hampshire, Kim Morley and Philip D. Marsh
*Centre for Applied Microbiology and Research, Salisbury, Wiltshire SP4 0JG, UK*

**Correspondence to:*
*Joanna Bacon, Centre for Applied Microbiology and Research, Salisbury, Wiltshire SP4 0JG, UK.*
*E-mail:*
*Joanna.bacon@camr.org.uk*

Received: 31 May 2002
Revised: 10 June 2002

### Abstract

**A unique approach, combining defined and reproducible *in vitro* models with DNA microarrays, has been developed to study environmental modulation of mycobacterial gene expression. The gene expression profiles of samples of *Mycobacterium tuberculosis*, from independent chemostat cultures grown under defined and reproducible conditions, were found to be highly correlated. This approach is now being used to study the effect of relevant stimuli, such as limited oxygen availability, on mycobacterial gene expression. A modification of the chemostat culture system, enabling large-volume controlled batch culture, has been developed to study starvation survival. Cultures of *M. tuberculosis* have been maintained under nutrient-starved conditions for extended periods, with $10^6$–$10^7$ bacilli surviving in a culturable state after 100 days. The design of the culture system has made it possible to control the environment and collect multiple time-course samples to study patterns of gene expression. These studies demonstrate that it is possible to perform long-term studies and obtain reproducible expression data using controlled and defined *in vitro* models. Copyright © 2002 John Wiley & Sons, Ltd.**

**Keywords:** ***Mycobacterium tuberculosis*; chemostat culture; latency; microarray; gene expression**

### Introduction

During infection, bacteria such as *Mycobacterium tuberculosis* encounter a dynamic host environment and modulate gene expression in order to adapt, survive and replicate. It is still unclear how *M. tuberculosis* responds to the stimuli it encounters *in vivo*, but post-genomic technologies have provided the tools and opportunity to study this interaction at the molecular and biochemical level [1,4,5,11].

We are using microarray technology to investigate environmental modulation of mycobacterial gene expression. Of particular interest is oxygen availability, as it is essential for the growth of *M. tuberculosis* and *in vitro* studies suggest that oxygen deprivation triggers the bacillus to enter a non-replicating persistent state, which is analogous to latency [3,9,10].

To gain maximum benefit from microarray experiments it is essential to study organisms from relevant and/or defined environments. This can be achieved by recovering tubercle bacilli from macrophages or from infected tissues; however, progress is restricted by poor recovery of bacteria. *In vitro* models of bacterial growth provide an alternative approach for environmental studies, provided the growth environment is controlled, defined and reproducible so that patterns of gene expression can be associated with distinct phenotypic states.

To investigate gene expression during two distinct stages of infection, viz. the active and latent stages, we developed two specialist culture systems; a chemostat to study replicating bacilli and a controlled batch culture system to study starvation survival. Chemostat culture allows bacteria to be grown continuously in a controlled and defined environment at a constant growth

Copyright © 2002 John Wiley & Sons, Ltd.

rate [8]. Using this technique, growth parameters can be varied separately and independently to study cause-and-effect relationships [6]. Chemostat culture, however, cannot be used to model starvation survival, as the bacilli enter a non-replicating persistent state. It was therefore necessary to modify the chemostat and develop a large-scale batch culture system which would allow us to monitor and control environmental conditions and collect multiple samples over time.

### Chemostat culture to study gene expression in replicating bacilli

A chemostat culture model was developed as detailed previously [7]. The culture system consisted of a 1 L fermentation vessel and was designed to facilitate continuous monitoring and control of temperature, pH, dissolved oxygen tension (DOT) and agitation. Continuous growth was achieved at a constant mean generation time of 24 h by controlling and balancing the rate of nutrient addition and effluent removal from the system. Using this culture system we have demonstrated that it is possible to grow dispersed cultures of *M. tuberculosis* continuously, at a constant mean generation time of 24 h, for 2–3 months under defined and reproducible conditions [7].

### Reproducibility of expression profiles

As a prerequisite for using this culture system to study environmental modulation of gene expression, it was necessary to establish the level of reproducibility of the gene expression profile from replicate cultures. Four independent cultures of *M. tuberculosis* strain H37Rv were established under aerobic carbon-limited conditions at a dissolved oxygen tension (DOT) of 50% air saturation, pH 6.9 and 37 °C. During steady-state growth, 10 ml aliquots of culture were collected from the culture system and were inactivated in 4 M guanidium thiocyanate (final concentration). Total RNA extracted with phenol:chloroform was used as a template to generate cy5-labelled cDNA. Genomic DNA was extracted from *M. tuberculosis* H37Rv using the procedure of Belisle and Sonnenberg, and was used to prepare cy3-labelled DNA [2].

Gene-specific whole genome arrays of *M. tuberculosis* H37Rv were prepared as detailed at **www.sghms.ac.uk/depts/medmicro/bugs**. Each array was hybridized with cy5-labelled cDNA and cy3-labelled DNA. Three arrays were performed on each of the four independent cultures, giving a total of 12 arrays. To maintain consistency between slides, one batch of genomic DNA was isolated and used to prepare the control sample for all the arrays.

Log ratio values for each spot (logarithmic in base 2 of cy5 over cy3), calculated using raw data, were averaged across the arrays performed on each culture. The correlation between the rankings of the average log ratio values was determined using the Spearman rank correlation (**www.cran.r-project.org**). The correlation between cultures was high, with all rank correlation coefficients being greater than 0.87. ANOVA confirmed that the variability between cultures was marginally less than the variability between arrays, and the largest remaining source of variation probably stemmed from using different scanning levels for cy3 and cy5 (data not shown).

### Future chemostat studies

Having established that the expression profiles of samples from independent chemostat cultures of *M. tuberculosis* are highly reproducible, this approach is now being used to study the effect of changes in oxygen tension on growth and gene expression. Cultures of *M. tuberculosis* have been grown at 50% and 1% DOT and the expression profiles are currently being compared and analysed. These studies have demonstrated that *M. tuberculosis* grows optimally at low oxygen tension and the major challenge at this stage is understanding the biological significance of the gene expression profiles and their relevance to pathogenesis.

### Batch culture model of starvation-survival

To study the behaviour of *M. tuberculosis* under starvation survival conditions, an adaptation of the chemostat culture system was used. The fermentation vessel was used as a closed batch-culture system while controlling and monitoring temperature, DOT, pH and agitation within the culture.

Copyright © 2002 John Wiley & Sons, Ltd. 

Using this culture system, separate 750 ml cultures of *M. tuberculosis* strain H37Rv were established under aerobic carbon-restricted and low-oxygen carbon-restricted conditions. Both cultures were established in Middlebrook 7H9 medium with ADC supplement and were allowed to proceed to exponential batch phase. For the aerobic carbon-restricted model, the DOT was maintained at 50% air saturation and the culture was allowed to grow through to an extended stationary phase for approximately 100 days. To achieve low-oxygen carbon-restricted growth, the DOT of the culture was lowered from 50% to 1% in a stepwise manner over a 5 day period from the point of mid-exponential growth. The DOT of the culture was maintained at 1% air saturation by sparging it with air or nitrogen as it progressed to extended stationary phase.

Using this approach, we have maintained cultures of *M. tuberculosis* under nutrient-starved conditions for extended periods, with $10^6$–$10^7$ bacilli surviving in a culturable state after 100 days. The design of the culture system has made it possible to control the temperature and DOT, and collect multiple time course samples to study physiology, biochemistry and patterns of gene expression. The deliberate use of a large-volume culture has also made it possible to avoid sample-to-sample variability associated with the use of small-volume cultures. Analysis of time course data is currently under way to identify genes correlating with survival.

Changes in the physiology of the bacterium as it adapted to starvation conditions have been responsible for experimental difficulties encountered during the course of this study. In particular, changes in the cell wall composition have affected the efficiency of the extraction protocol and the resultant RNA yield. Extraction protocols have been modified to incorporate multiple chloroform extractions and this has improved RNA recovery. RNA yield is also likely to have been affected by the decline in cell viability during survival, as less than 1% of the culture remained viable by day 100.

These studies demonstrate the value of *in vitro* models for studying the effect of relevant stimuli and for associating patterns of gene expression with specific stimuli and phenotypes. By paying close attention to the control and design of the model, it is possible to perform long-term studies, obtain plentiful RNA and obtain highly reproducible expression data. The use of DNA as a reference across all arrays has also introduced a unifying control element into all arrays and will enable us to compare the expression profiles generated in response to different environmental stimuli. It is envisaged that these studies, which identify the strategies adopted when responding to environmental stimuli, will ultimately lead to a more complete understanding of how *M. tuberculosis* interacts with the host.

### Acknowledgements

We acknowledge BμG@S (the Bacterial Microarray Group at St George's) and The Wellcome Trust for funding their work. We also thank Lorenz Wernisch, School of Crystallography, Birkbeck College, University of London, UK, for statistical analysis of data. This work was funded by the Department of Health (UK) and The Wellcome Trust.

### References

1. Barry CE, Schroeder BG. 2000. DNA microarrays: translational tools for understanding the biology of *Mycobacterium tuberculosis*. *Trends Microbiol* **8**: 209–210.
2. Belisle JT, Sonnenberg MG. 1998. Isolation of genomic DNA from mycobacteria. *Methods Mol Biol* **101**: 31–44.
3. Cunningham AF, Spreadbury CL. 1998. Mycobacterial stationary phase induced by low oxygen tension: cell wall thickening and localization of the 16-kDa α-crystallin homolog. *J Bacteriol* **180**: 801–808.
4. DeRisi JL, Iyer VR, Brown PO. 1997. Exploring the metabolic and genetic control of gene expression on a genomic scale. *Science* **278**: 680–686.
5. Harrington CA, Rosenow C, Retief J. 2000. Monitoring gene expression using DNA microarrays. *Curr Opin Microbiol* **3**: 285–291.
6. James BW, Mauchline WS, Fitzgeorge RB, *et al.* 1995. Influence of iron-limited continuous culture on physiology and virulence of *Legionella pneumophila*. *Infect Immun* **63**: 4224–4230.
7. James BW, Williams A, Marsh PD. 2000. The physiology and pathogenicity of *Mycobacterium tuberculosis* grown under controlled conditions in a defined medium. *J Appl Microbiol* **88**: 669–677.
8. Pirt SJ. 1975. *Principles of Microbe and Cell Cultivation*. Blackwell Scientific: Oxford.
9. Sherman DR, Voskuil M, Schnappinger D, *et al.* 2001. Regulation of the *Mycobacterium tuberculosis* hypoxic response gene encoding α-crystallin. *Proc Natl Acad Sci USA* **98**: 7534–7539.
10. Wayne LG, Hayes LG. 1996. An *in vitro* model for sequential study of shiftdown of *Mycobacterium tuberculosis* through two stages of non-replicating persistence. *Infect Immun* **64**: 2062–2069.
11. Wilson M, DeRisi J, Kristensen HH, *et al.* 1999. Exploring drug-induced alterations in gene expression in *Mycobacterium tuberculosis* by microarray hybridization. *Proc Natl Acad Sci USA* **96**: 12 833–12 838.

Copyright © 2002 John Wiley & Sons, Ltd. 
